# Chinese and Global Distribution of H9 Subtype Avian Influenza Viruses

**DOI:** 10.1371/journal.pone.0052671

**Published:** 2012-12-21

**Authors:** Wenming Jiang, Shuo Liu, Guangyu Hou, Jinping Li, Qingye Zhuang, Suchun Wang, Peng Zhang, Jiming Chen

**Affiliations:** 1 The Laboratory of Avian Disease Surveillance, China Animal Health and Epidemiology Center, Qingdao, China; Friedrich-Loeffler-Institut, Germany

## Abstract

H9 subtype avian influenza viruses (AIVs) are of significance in poultry and public health, but epidemiological studies about the viruses are scarce. In this study, phylogenetic relationships of the viruses were analyzed based on 1233 previously reported sequences and 745 novel sequences of the viral hemagglutinin gene. The novel sequences were obtained through large-scale surveys conducted in 2008-2011 in China. The results revealed distinct distributions of H9 subtype AIVs in different hosts, sites and regions in China and in the world: (1) the dominant lineage of H9 subtype AIVs in China in recent years is lineage h9.4.2.5 represented by A/chicken/Guangxi/55/2005; (2) the newly emerging lineage h9.4.2.6, represented by A/chicken/Guangdong/FZH/2011, has also become prevalent in China; (3) lineages h9.3.3, h9.4.1 and h9.4.2, represented by A/duck/Hokkaido/26/99, A/quail/Hong Kong/G1/97 and A/chicken/Hong Kong/G9/97, respectively, have become globally dominant in recent years; (4) lineages h9.4.1 and h9.4.2 are likely of more risk to public health than others; (5) different lineages have different transmission features and host tropisms. This study also provided novel experimental data which indicated that the Leu-234 (H9 numbering) motif in the viral hemagglutinin gene is an important but not unique determinant in receptor-binding preference. This report provides a detailed and updated panoramic view of the epidemiological distributions of H9 subtype AIVs globally and in China, and sheds new insights for the prevention of infection in poultry and preparedness for a potential pandemic caused by the viruses.

## Introduction

Influenza viruses in the family *Orthomyxoviridae* are classified into three types, A, B, and C. Type A influenza viruses are further classified into different subtypes based on antigenic differences in the viral surface glycoproteins, hemagglutinin (HA) and neuraminidase (NA). Currently, 16 HA subtypes (H1−H16) and 9 NA subtypes (N1−N9) of influenza viruses have been isolated from birds, and a novel HA subtype (H17) and a novel NA subtype (N10) of influenza viruses have been identified in bats [1]. Among the 16 HA subtypes of avian influenza viruses (AIVs), H9 subtype (mainly H9N2 subtype) in domestic fowls and wild birds have been reported in various regions including North America, Europe, Asia, Africa and the Pacific [2]–[9].

Usually, H9 subtype AIVs cause mild clinical signs in birds; however, the infection can be exacerbated by a secondary bacterial infection [10]–[21]. In recent years, H9 subtype AIVs circulating in China aroused worldwide concerns, because several cases of human infections in China have been identified since the end of the 1990s, and some of the viruses displayed human influenza virus-like receptor specificity [11], [22]–[29]. H9 subtype AIVs viruses are thus considered one of the most likely candidates to cause a new influenza pandemic in humans [22]–[27]. Unlike human infection with the H5 subtype highly pathogenic avian influenza (HPAI) virus that usually causes a severe infection, the associated disease symptoms in all human cases of H9 subtype AIV infection have been mild, with no evidence of human-to-human transmission [22]–[27], [30].

As an essential component of the global strategy for pandemic preparedness, selection of candidate H5 and H9 influenza vaccine viruses has been coordinated by the World Health Organization (WHO) in recent years, through epidemiological surveys and phylogenetic analysis [25], [26]. However, as clearly stated in two relevant reports issued by the WHO in 2011, data characterizing recent H9 subtype AIVs circulating in the world are limited, and the majority of the viruses that have been sequenced belong to the G1 clade, represented by A/quail/Hong Kong/G1/97, or the Y280/G9 clade, represented by A/duck/Hong Kong/Y280/97 or A/chicken/Hong Kong/G9/97 [25], [26].

The goal of this study was to elucidate the host, spatial and phylogenetic distributions of H9 subtype AIVs circulating in China and in the world in recent years, through epidemiological surveys and phylogenic analysis of viral HA gene sequences. This viral gene is vital for viral pathogenicity, antigenicity and host range, although some evidence indicates that these traits are polygenic [31].

## Results

### Swab Sample Detection

A total of 31406 swab samples were collected from 299 live bird markets, 26 slaughtering houses, 303 backyard flocks and 348 poultry farms through the eight surveys conducted in 2008−2011. Among them, 57 were positive for Newcastle disease viruses (NDVs), and 1349 were positive for AIVs. Among the 1349 AIV positive samples, 950 were positive for H9 subtype AIVs and 76 were positive for H5 subtype AIVs. The prevalence of H9 subtype AIVs in chicken samples, 3.50% (884/25233), was significantly higher than the prevalence in samples from waterfowl including ducks and geese, 1.08% (66/6127), with *P*<0.05. None of the 46 pigeon samples were positive for AIVs. The prevalence of H9 subtype AIVs in the samples from live bird markets, 6.84% (655/9558), or in the samples from slaughtering houses, 3.81% (47/1232), was significantly higher than that in the samples from backyard flocks, 1.82% (172/9464), or from poultry farms, 0.68% (76/11152), all with *P*<0.05. On average, infection of H9 subtype AIVs was identified in 35.12% (105/299) of the sampled live bird markets, 34.62% (9/26) of the sampled slaughtering houses, 17.49% (53/303) of the sampled backyard flocks, and 11.21% (39/348) of the sampled poultry farms. The prevalence of H9 subtype AIVs in Northern China, 2.85% (350/12273), was similar to its counterpart in Southern China, 3.14% (600/19133), with *P*>0.05.

### Panorama of the Global Phylogenetic Distribution of H9 Subtype AIVs

We analyzed the sequence phylogenetic relationships of this report using the neighbor-joining method and the maximum likelihood method (G+I), and found the results of the two methods were of little difference. Therefore, only the results of the neighbor-joining method were presented.

As of September 17, 2011, HA sequences (>900 bp) of 1233 H9 subtype AIVs with clear background were available in GenBank, namely that the source, isolation place and isolation time of the sequences were available and they have not been suspected by any publications [14], [16]. Of these, 55 were reported by our laboratory. The remaining 1178 sequences were submitted by other research groups.

Phylogenetic analysis of the 1178 sequences revealed four primary lineages, h9.1−h9.4, some secondary and tertiary lineages (Figure S1). The temporal and spatial distribution of the lineages is showed in Table S1and Figure S2.

Genetic distances in the HA1 subunit of the viral HA gene among the lineages are showed in Table S2. In general, the genetic distances were 20.00% to 24.39% between primary lineages, 11.93% to 17.27% between secondary, and 5.81% to 18.28% between tertiary lineages.

At the primary lineage level, lineages h9.1 and h9.2 corresponded to only two viruses isolated in 1966 in North America and three viruses isolated in the 1990s in North America, respectively. Lineage h9.3 corresponded to 140 viruses isolated in the Eastern Hemisphere, and 33 viruses isolated in North America. Lineage h9.4 corresponded to hundreds of viruses from the Eastern Hemisphere. Though lineage 9.4 harbored many more sequences than lineage h9.3, the latter was more widely distributed, with viruses from Asia, Europe, Africa, the Pacific and North America, while lineage h9.4 circulated exclusively in Asia. Lineage h9.3 also had a longer circulation history (1976−2010) than lineage h9.4 (1994−2011).

At the secondary lineage level, most avian H9N2 viruses isolated in recent years in China, and those isolated in the Asian countries west to China including Pakistan, India, Iran, and Israel, belonged to the secondary lineages h9.4.2 and h9.4.1, respectively. Previously, these two secondary lineages were designated as the Y280/G9-like and G1-like viruses, respectively [25], [26]. Lineage h9.4.2 comprised more sequences than lineage h9.4.1, which further comprised more sequences than lineage h9.3.3 represented by A/duck/Hokkaido/26/99. However, lineage h9.3.3 was more widely distributed than h9.4.2 and h9.4.1, with isolates from Asia, Europe, Africa, the Pacific and North America. Lineages h9.4.1 circulated almost exclusively in Asian countries and h9.4.2 in China. In addition, lineages h9.3.3, h9.4.1 and h9.4.2 had a similar circulation history, from approximately the 1990s to the present.

At the tertiary lineage level, lineage h9.3.3.2 was distributed more widely than the other tertiary lineages with isolates from Asia, Europe, Africa, the Pacific and North America.

Taken together, at the primary, secondary and tertiary lineage levels, lineages h9.3, h9.3.3, h9.3.3.2 should be globally dominant compared to their counterparts. In addition, lineages h9.4.1 and h9.4.2, namely the G1-like and Y280/G9-like viruses, were also assumed to be globally dominant because they were prevalent in many countries in Asia and in many provinces of China (a country covering a large land area), respectively.

### Host Distribution and Transmission of Different H9 Subtype AIVs

Most (>90%) of the viruses in lineages h9.4.1, h9.4.2 and h9.3.3.1 were from domestic terrestrial birds like chickens and quails, while most (>90%) in lineages h9.3.1 and h9.3.3.2 were from domestic or wild waterfowl (Figure S1). Furthermore, most branches within lineages h9.4.1, h9.4.2 and h9.3.3.1, namely those mainly from domestic terrestrial birds, covered viruses from one country only, indicating that transboundary transmission was infrequent within these lineages. In contrast, most branches in lineages h9.3.1 and h9.3.3.2, namely those mainly from domestic or wild waterfowl, covered viruses from many countries, indicating that transboundary transmission was frequent within these lineages (Figure S2).

### Phylogenetic Distribution of H9 Subtype AIVs in China

Among the 1178 H9 subtype AIVs, 833 were from China including Hong Kong SAR with a clear background. These 833 sequences were reported by other research groups. As shown in Table S1 and Figure S2, among these 833 viruses, 767 from dozens of provinces in China belonged to lineage h9.4.2, 48 from Hong Kong and a city in Guangdong Province, (Shantou) belonged to lineage h9.4.1, and the remaining 18 belonged to other lineages including h9.3.1, h9.3.3 or h9.3.2. Therefore, lineage h9.4.2 was more dominant than lineage h9.4.1 in China.

As shown in Table S1 and Figure S2, 88.42% (504/570) of the viruses in lineages h9.4.2.1−h9.4.2.4 were isolated before 2007, while 75.65% (166/193) in lineage h9.4.2.5, represented by A/chicken/Guangxi/55/2005, were isolated in dozens of provinces in China in 2007−2011, while lineage h9.4.2.6, represented by A/chicken/Guangdong/FZH/2011, comprised 26 viruses isolated in the provinces of Fujian and Guangdong in China in 2010−2011. Therefore, lineage h9.4.2.5 was more dominant in China in recent years than other tertiary lineages.

We obtained the HA gene sequences of 800 H9 subtype AIVs, through an unpublished pioneer survey in 2007 and the eight surveys reported here. Among these 800 sequences, 302 were not fully identical to any others. In addition, among these 800 sequences, 55 were published previously [14], and the remaining 745 ones were first reported herein. Their GenBank accession numbers were JN802529−JN802662, JN803947−JN804557, FJ434561−FJ434587, FJ807708−FJ807719, and GQ463212−GQ463227. As shown in Figure S3, 769 of the 800 viruses belonged to lineage h9.4.2.5, and these were isolated in dozens of provinces during 2007−2011. Another 22 viruses belonged to lineage h9.4.2.6 and these were isolated in five provinces (two in South China, one in Southeast China, one in East China and one in Northwest China) in 2011. The remaining nine viruses belonged to lineage h9.4.2.4, and were isolated in one province in 2008. Therefore, our data were consistent with the suggestion of Figure S1 using the sequences reported by other research groups. Furthermore, our data indicated that lineage h9.4.2.6 had also been prevalent in China in 2011, as it was identified from South, Southeast, East, and Northwest China.

We separated the phylogenetic analysis of the HA gene sequences of H9 subtype AIVs reported by others and us, in order to minimize potential analytical bias caused by so many HA gene sequences reported by us.

### Phylogenetic Distribution of H9 Subtype AIVs in Countries Near to China

Among the 1178 viruses, all the 65 H9 subtype AIVs from Korea belonged to h9.3, distinct from the dominant lineage h9.4 in China. Similarly, all the viruses from Vietnam, Pakistan and India were distinct from the dominant lineages circulating in China in the corresponding periods. Although H9 subtype AIVs in lineage 9.4.2 have been widely circulating in China for years, only a few viruses within the lineage were isolated in another country, Japan. This indicated that the transboundary spreading of H9 subtype between China and its neighboring countries occurred infrequently. This is consistent with the finding that transboundary transmission was infrequent within the lineages mainly from domestic terrestrial birds, because the viruses from China and its neighboring countries belonged to lineages mainly from domestic terrestrial birds.

In Asian countries west to China including Pakistan, India, Iran, and Israel, h9.4.1 has been dominant in recent years. At the tertiary lineage level, as partially shown in Figure S2 and Table S1, lineage h9.4.1.5 has been dominant over others in these Asian countries since 2007, based on the limited sequences available in GenBank.

### Antigenic Analysis Using the HI Assay

Antigenic cross-reactivity of randomly selected viruses was investigated by the HI assay. As showed in Table 1, the results suggested that the viruses in lineages h9.4.2.3 and h9.4.2.4 did not react well to the chicken antisera against the viruses in lineages h9.4.2.5 and h9.4.2.6, although viruses in lineages h9.4.2.5 and h9.4.2.6 reacted well to the antisera against viruses in lineages h9.4.2.3 and h9.4.2.4, indicating that lineages h9.4.2.5 and h9.4.2.6 have become somehow antigenically distinct from lineages h9.4.2.3 and h9.4.2.4.

**Table 1:** Homologous and heterogeneous hemagglutination inhibition titers of some H9 lineages^a^.

Antisera	Antigen						
	NX184 (h9.4.2.6)	GB26 (h9.4.2.6)	AK4 (h9.4.2.5)	AE15 (h9.4.2.5)	YB06 (h9.4.2.4)	BZ02 (h9.4.2.3)	GX10 (h9.4.2.3)
NX184 (h9.4.2.6)	**64**	16	32	16	4	4	4
GB26 (h9.4.2.6)	64	**64**	64	32	2	4	4
AK4 (h9.4.2.5)	64	64	**128**	128	16	8	32
AE15 (h9.4.2.5)	32	32	128	**128**	8	8	16
YB06 (h9.4.2.4)	128	64	128	64	**128**	32	64
BZ02 (h9.4.2.3)	64	64	64	16	32	**64**	64
GX10 (h9.4.2.3)	32	32	64	64	16	16	**64**

a Homologous titers are in boldface; lineage designations are in parentheses; virus designations are abbreviated as follows: NX184 = A/chicken/Ningxia/NX184/2011, GB26 = A/chicken/Guizhou/GB26/2011, AK4 = A/chicken/Anhui/AK4/2011, AK15 = A/chicken/Anhui/AK15/2011, YB06 = A/chicken/Shandong/YB06/2006, BZ02 = A/chicken/Shandong/BZ02/2002, GX10 = A/chicken/Guangxi/10/1999.

### Prevalence of the Leu-234 Motif in the Viral HA Gene

Leu-234 (corresponding to residue 226 in H3 numbering) at the receptor-binding site (RBS) in the HA gene of H9 subtype AIVs likely has human influenza virus-like receptor specificity [24], [32]–[35]. Among the 1178 H9 subtype AIVs (Figure S2), none of the 178 viruses in lineages h9.1, h9.2 and h9.3 carried the motif Leu-234, while nearly 75% of the viruses in lineages h9.4.1 (165/222) and h9.4.2 (582/778) carried the motif.

### Analysis of Receptor-binding Preference Using the HA Assay

All the five NDVs and the two human influenza viruses reacted well with the untreated goose RBCs and α2,3-specific sialidase-treated goose RBCs with no difference in HA titers. This indicated that the viruses did not bind to α2,3-linked sialic acid on the surface of the goose RBCs. All the ten H5 subtype avian influenza viruses reacted only with untreated goose RBCs, indicating that they bound only to α2,3-linked sialic acid on the surface of the goose RBCs. The 76 H9 subtype AIVs (72 in lineage h9.4.2.5, 3 in lineage h9.4.2.6, 1 in lineage h9.4.2.3) reacted well with untreated goose RBCs, but their HA titers using treated goose RBCs declined to 0−100% of their counterparts using untreated goose RBCs (Table S3). This indicated that these H9 subtype AIVs had different affinities for binding to α2,6-linked sialic acid. Of the 76 H9 subtype viruses, 55 (54 carrying the Leu-234 motif) reacted with untreated and treated goose RBCs, indicating that they could bind to α2,6-linked sialic acid on the surface of the goose RBCs. The remaining 21 (20 carrying the Leu-234 motif) viruses reacted only with untreated goose RBCs and not treated goose RBCs, indicating that they bound only to α2,3-linked sialic acid on the surface of the goose RBCs.

## Discussion

As mentioned in the introduction of this report, epidemiological studies on distribution of H9 subtype AIVs in some countries have been reported previously. However, most of them covered limited sequences or limited regions, not at Chinese national level or global level. This report presents a detailed and updated panoramic view of the distribution of H9 subtype AIVs globally and in China, partially based on 745 novel HA gene sequences of H9 subtype AIVs obtained through large-scale surveys. This is also the first report using surveillance data to indicate the wide existence of the new lineage h9.4.2.6 in China and the viral prevalences in different sites in China. In addition, this report provides novel experimental data to show that the Leu-234 motif in the viral HA gene is not a unique determinant in receptor-binding preference for α2,6-linked sialic acid.

The global views of H9 subtype AIVs reported here reflected only a part of the reality, mainly because relevant data are scarce for many countries and regions. Lineages of this report were classified mainly according the genetic distances and the topology of relevant phylogenetic trees. It should be noted that the bootstrap values of some lineages including h9.4.1.3, h9.4.2.3 and h9.4.2.4 in Figure S1 were not high. This may result from that some intermediate strains were covered in the analysis, as they, in nature, could not be assigned to any lineages with confidence. Similar phenomena have been observed in our previous phylogenetic analysis of other influenza A and influenza B viruses [36], [37]. Furthermore, also due to the existence of some intermediate, some viruses in a tertiary lineage were of genetic distances longer than the genetic distances of some pairs between the secondary lineages. Similar phenomena have been observed in phylogenetic analysis of subtype H5 avian influenza A viruses, e.g. some viruses in clade 2.3.2 were of genetic distances longer than some pairs between clade 2.3.2 and clade 2.3.4 [38].

This report suggests that some lineages of H9 subtype AIVs are more prevalent in terrestrial birds including chickens and quails than in waterfowl, e.g. >90% of the viruses in lineages h9.4.1, h9.4.2 and h9.3.3.1 in Figure S1 were from domestic terrestrial birds. These lineages may have adapted to terrestrial birds rather than to waterfowl. Furthermore, the distinct host tropisms of the viruses have likely led to different frequencies of transboundary transmission, as domestic and wild waterfowl frequently share open water bodies which can facilitate the transmission of the viruses. In addition, wild waterfowl can also transmit viruses into another country via migration.

The surveys revealed that the prevalence of H9 subtype AIVs was similar in Southern and Northern China. This is different from the distribution of H5 subtype HPAI viruses [39]. This might be because the H9 subtype lineages dominant in China have become more adapted to terrestrial birds. Thus, the ecological features of Southern China with prevalent open water bodies and waterfowl, which facilitate the transmission of H5 subtype HPAI viruses, do not facilitate the transmission of H9 subtype viruses.

Live poultry markets have a high prevalence of infection with H5 subtype HPAI viruses in some countries [39], [40]. We also found that the prevalence of H9 subtype of AIVs in live poultry markets was significantly higher than in backyard flocks and poultry farms, suggesting that live poultry markets are important for transmission of multiple subtypes of AIVs.

The surveys indicated that, on average, infection of H9 subtype AIVs was identified in 35.12% of the sampled live bird markets, 17.49% of the sampled backyard flocks, and 11.21% (39/348) of the sampled poultry farms. These data suggested that the viruses are widely circulating in poultry in China. Recent reports also indicated that the viruses were also widely circulating in many other countries or regions [2−5], [7−12], [15], [17], [20], . For example, in Jordan, a survey suggested that more than 50% of broiler or layer flocks were carrying H9 subtype AIVs [18]. In South Korea, a survey suggested that 8.09% of fecal samples of ducks and chickens collected from live poultry markets were H9 subtype AIVs positive [19].

The avian influenza reports issued by the WHO in 2010 and 2011 all suggested that lineages h9.4.1 and h9.4.2 have been globally dominant in recent years [25], [26]. This report provides evidence through phylogenetic analysis that another lineage, h9.3.3.2, is also globally dominant.

Lineage h9.4.2, or the Y280/G9-like clade, has been dominant in China in recent years; however, we found that the lineage recently evolved into two main lineages, h9.4.2.5 and h9.4.2.6. Table S2 indicates that lineages h9.4.2.5 and h9.4.2.6 have become genetically distinct from their precedent lineages h9.4.2.3 and h9.4.2.4 which have largely disappeared in China. Therefore, in principle, it should be better to select the vaccine strains used for prevention of the viral infection in poultry from lineages h9.4.2.5 and h9.4.2.6. However, most of the current H9 subtype AIVs used for the production of inactivated vaccines in China, such as A/chicken/Guangdong/SS/94, A/chicken/Shandong/6/96, A/chicken/Shanghai/F/98, A/chicken/Guangxi/10/99, belong to lineages h9.4.2.3 or h9.4.2.4. Interestingly, as showed in Table 1, although viruses from the earlier lineages h9.4.3.3 and h9.4.3.4 (YB06, BZ02 and GX10) did not react well with antisera raised against viruses from the currently prevalent lineages h9.4.3.5 and h9.4.3.6, viruses from lineages h9.4.3.5 and h9.4.3.6 still reacted well with antisera raised against the earlier strains. Therefore, whether antibodies raised by vaccination can protect a host from infection of currently circulating strains should be evaluated in the future by neutralization tests and/or animal infection experiments, rather than the HI assays only.

It has been reported that the Leu-234 motif in the HA gene of H5 and H9 subtype influenza viruses is an important determinant in receptor-binding preference for α2,6-linked sialic acid [24], . This was supported by our analysis of receptor-binding preference using α2,3-specific sialidase-treated goose RBCs, because most (54/76) of the viruses carrying the Leu-234 motif bound to α2,6-linked sialic acid on the surface of α2,3-specific sialidase-treated goose RBCs. According to the distribution of the Leu-234 motif in the viral HA gene, this report indicates that lineages h9.4.1 and h9.4.2 are of greater risk to public health than others. Therefore, in principle, the vaccine strains for pandemic preparedness should be selected from lineages h9.4.1 and h9.4.2. Favorably, they should be from the tertiary lineages h9.4.2.5 and h9.4.1.5, which are likely to be dominant in China and the Asian countries west to China, respectively. Although lineage h9.3.3.2 is also globally dominant, it seemed to be less of a threat to public health, because no viruses in this lineage carrying the Leu-234 motif. In addition, all the 11 H9 subtype AIVs identified from humans so far belonged to lineage h9.4.1 or lineage h9.4.2 (Figure S2), except one (A/Korea/KNBP-0028/2000) from lineage h9.3.3.1.

Interestingly, this report also indicates that the Leu-234 motif is not a unique determinant in receptor-binding preference for α2,6-linked sialic acid, because a few (20/76) H9 subtype viruses carrying the Leu-234 motif did not react with α2,3-specific sialidase-treated goose RBCs, and one virus not carrying the Leu-234 motif reacted well with α2,3-specific sialidase-treated goose RBCs (Table S3). Previous reports also indicated that some other residues, like those at sites 191, 197, 198 and 238, may also affect cell tropism [34], [35].

Taken together, this report presents an updated, detailed and panoramic view of the recent epidemiological distribution of H9 subtype AIVs in China and around the world, and sheds new insights for prevention of the infection in poultry and preparedness for a potential pandemic caused by the viruses.

## Materials and Methods

### Animal Welfare

This study was conducted according to the animal welfare guidelines of the World Organization for Animal Health [41], and approved by the Animal Welfare Committee of China Animal Health and Epidemiology Center.

### The Hemagglutination (HA) and Hemagglutination Inhibition (HI) Assays

The HA and HI assays were performed using 96-well U-bottom microtiter plates as described previously [42]. For the HA assay, each virus stock to be tested was diluted serially in the plates using phosphate-buffered saline (PBS, pH 7.2). Thereafter, 0.5% (v/v) chicken or goose RBCs were added to each well, and the plates were incubated at 4°C for 45−60 min. The HA titer was the inverse of the last dilution of the virus stock showing complete agglutination of the RBCs. For the HI assay, each antiserum to be tested was serially diluted in the plates using PBS, and then four hemaglutination unit (HAU) of working solution of standard viral antigen was added to each well of the plates, except those served as serum and RBC controls. The plates were incubated at 37°C for 30 min. Thereafter, 0.5% (v/v) chicken or goose RBCs were added into all wells, and the plates were incubated at 4°C for 45−60 min. The HI titer was the inverse of the last dilution of sera showing complete inhibition of the RBC agglutination.

### Sampling and Virus Isolation

**Figure 1:**
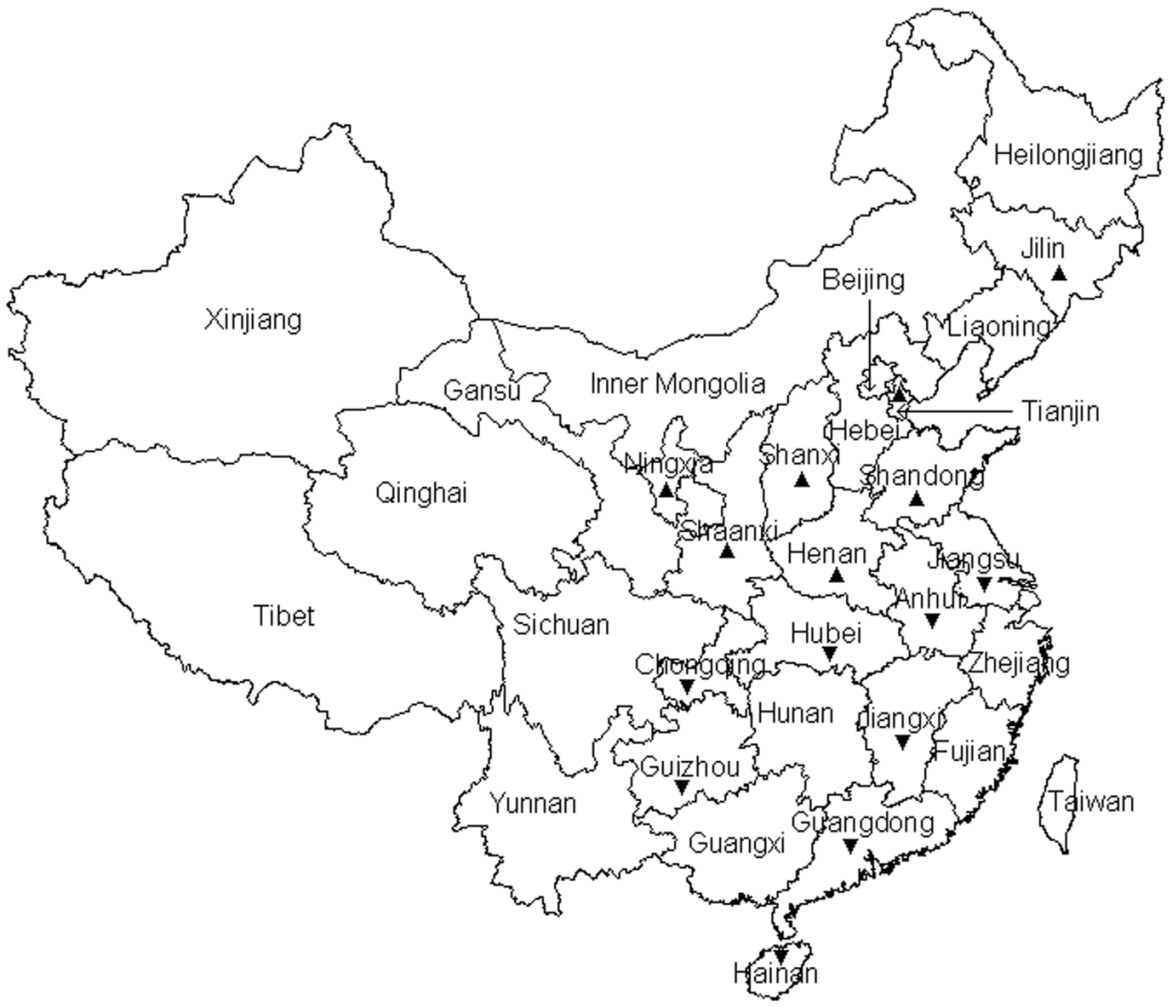
Provinces targeted for the eight surveys in 2008−2011 marked with “▴” for those in Northern China or “▾” for those in Southern China.

During 2008−2011, eight epidemiological surveys of AIVs were conducted twice a year. Each survey targeted 8−12 provinces (Figure 1), from which dozens of poultry farms, backyard flocks, slaughtering houses and live bird markets were selected at random. For each site, usually 30 birds were selected randomly for swab sample collection, and a total of 31406 swab samples were collected. Swab samples were collected by taking smears from the trachea and cloacae of the domestic fowls and placed in a transport medium. The samples were clarified by centrifugation at 10000 *g* for 5 min, and the supernatants were inoculated in 10-day-old specific-pathogen-free (SPF) chicken embryonated eggs via the allantoic sac route. The eggs were further incubated for four days, and checked twice each day during the incubation period. The dead ones were picked out and stored in a refrigerator. After the incubation period, the allantoic fluids of the live embryos were collected and tested by using the HA assay. All the hemagglutination-positive samples and the allantoic fluid of the dead eggs were investigated further by RT-PCR, as described below.

### Reverse Transcription-polymerase Chain Reaction (RT-PCR)

RNA was extracted using an RNeasy Mini Kit (Qiagen, Hilden, Germany), and amplified with the PrimeScript One-step RT-PCR Kit (TaKaRa, Dalian, China). Most amplifications used the primers 5′-CTCAGGGAGCAAAAGCAGGGG-3′ (upper) and 5′-GTATTAGTAGAAACAAGGGTG TTTT-3′ (down) which cover the entire length of the HA gene [43]. Some used the primers 5′-CCAAAGAATTGCTCCACACAGA-3′ (upper) and 5′-GCACAAGAGATGAGGCGACA GT-3′ (down) which flank a 1500 bp segment of the HA gene of H9 subtype AIVs. RT-PCR was performed in a 50-µL reaction using the One-Step RT-PCR kit (TaKaRa, Dalian, China), with incubation at 50°C for 30 min and denaturation at 94°C for 2 min, followed by 30 cycles at 94°C for 30 s, 55°C for 30 s and 72°C for 2 min. RT-PCR products were purified with an agarose gel DNA extraction kit (TaKaRa, Dalian, China) and ligated into the pGEM-T Easy vector (Promega, Shanghai, China). Positive clones were sequenced using a Perkin-Elmer model 377 XL DNA sequencer in both directions using the T7 and SP6 promoter primers. Subtypes of these sequences were determined using the BLAST tool in the Influenza Virus Resource [44]. Some RT-PCR positives targeting the 1500 bp segment of the H9 subtype AIV gene were not sequenced because they were from samples collected at the same time from the same site.

### Distribution Analysis

Distributions of the samples detected positive for H9 subtype AIVs through the RT-PCR and sequence analysis in hosts, sites and regions were analyzed according to their background information. Differences in prevalence were examined using a chi-square test, and *P*<0.05 was considered statistically significant. The sampled provinces in Northern and Southern China were shown in Figure 1. The Eastern Hemisphere covers Asia, Europe, Africa and the Pacific, while the Western Hemisphere means North and South America.

### Phylogenetic Analysis

All the HA sequences (≥900 bp) of H9 subtype AIVs that were available in GenBank were downloaded through the Influenza Virus Resource [44]. They were analyzed along with the viral HA sequences obtained through the surveys reported here using the software MEGA 5.05 (http://www.megasoftware.net/) [45]. Sequences were aligned using the software MUSCLE [46]. Genetic distances and phylogenetic relationships were calculated by the neighbor-joining method, based on the sequences of the HA1 subunit of the viral HA gene [47]. Nucleotide substitutions were set under the Kimura 2-parameter model, and substitution rates among sites were set in gamma distribution [47]. The gaps were handled by pairwise deletion. Bootstrap values were calculated from 1000 replicates.

### Numbering of Amino Acid Residues

Amino acid residues, unless noted otherwise, were numbered according to the HA sequence of A/chicken/Anhui/AK13/2008 (H9) with GenBank accession number FJ434582.

### Classification of Lineages and Sublineages

Lineages and sublineages were classified mainly according to genetic distances and topology of the phylogenetic trees, as described in two previous reports [14], [36]. Primary lineage designations began with the subtype name plus a point and a number, *e.g.,* h9.3 represents the third lineage of H9 subtype AIVs. Secondary lineage designations began with their primary lineage designations plus a point and a number, *e.g.*, h9.3.2 represents the second sublineage of lineage h9.3. The numbers within the lineage designations, if possible, were in order of Western Hemisphere followed by Eastern Hemisphere in geography, and in order of past to present in isolation time. Classification of the sublineages within lineage h9.3 was modified here, compared with a previous report [36], namely that lineage h9.3.2 in the previous report was designated h9.3.1.3 in this report, and h9.3.2 represented an unclassified lineage in the previous report.

### Antigenic Analysis Using the HI Assay

Antigenic cross-reactivity was investigated using the aforementioned HI assay. Polyclonal antibodies against seven randomly selected strains of H9 subtype AIVs (two in lineage h9.4.2.6, two in lineage h9.4.2.5, two in lineage h9.4.2.4, one in lineage h9.4.2.3) were generated using 21 day-old SPF chickens. Four of the seven strains, A/chicken/Ningxia/NX184/2011, A/chicken/Guizhou/GB26/2011, A/chicken/Anhui/AK4/2011 and A/chicken/Anhui/AK15/2011, were obtained through the surveys presented here. The remaining three, A/chicken/Shandong/YB06/2006, A/chicken/Shandong/BZ02/2002 and A/chicken/Guangxi/10/1999, were obtained from a biological company (Yebio, Qingdao), and the GenBank accession numbers of their HA gene sequences were AJ12345− AJ12348. Each of the SPF chickens was immunized with 0.5 mL virus-infected allantoic fluid inactivated using 0.2% (v/v, final) formaldehyde through intramuscular injection for only one time, and antisera were collected on the 21st day after the injection.

### Analysis of Receptor-binding Preference Using the HA Assay

Receptor-binding preference of 76 H9 subtype AIVs, ten H5 subtype AIVs, five NDVs, and two human influenza viruses was examined using the HA assay with untreated goose RBCs and α2,3-specific sialidase-treated goose RBCs, according to a previous report [48]. All the 93 avian influenza or NDVs were isolated from the samples collected through this study, except the aforementioned two, A/chicken/Shandong/YB06/2006 and A/chicken/Shandong/BZ02/2002. The human influenza viruses, A/Qingdao/1038/2007(H3N2) and A/Qingdao/783/2007(H1N1), were obtained from Qingdao Center for Disease Control. For sialidase treatment, a 10% (v/v) suspension of goose RBCs was prepared in PBS and a 100 µl aliquot treated with 1.25 U of recombinant α2,3-sialidase (Takara, Japan) for 1 h at 37°C. Treated erythrocytes were washed twice and adjusted to a final working concentration (0.5%, v/v) with PBS. With sialidase treatment, α2,3-linked sialic acid was completely eliminated from the surface of goose RBCs without damaging α2,6-linked sialic acid.

## Supporting Information

Figure S1 **Small version of the phylogenetic tree of 1178 H9 subtype AIVs based on HA1 subunit of the viral hemagglutinin gene sequences.** Lineage designations are to the right of relevant branches, and bootstrap values are at relevant nodes. The representative virus of each lineage is marked with an asterisk. This figure is too large, and it actual size can be viewed using Windows-Picture-&-Fax Viewer via pressing the keys “Ctrl” and “A” simultaneously.
(TIF)

Figure S2 **Phylogenetic distribution of 1178 H9 subtype AIVs in GenBank based the sequences of the HA1 subunit of the viral hemagglutinin gene.** Lineage designations are to the right of the relevant branches, and bootstrap values are at relevant nodes. This figure is too large, and it actual size can be viewed using Windows-Picture-&-Fax Viewer via pressing the keys “Ctrl” and “A” simultaneously.
(TIF)

Figure S3 **Phylogenetic distribution of 800 H9 subtype AIVs based the sequences of the HA1 subunit of the viral hemagglutinin gene.** The representative virus of each lineage is marked with an asterisk. Lineage designations are to the right of relevant branches, and bootstrap values are at relevant nodes. This figure is too large, and it actual size can be viewed using Windows-Picture-&-Fax Viewer via pressing the keys “Ctrl” and “A” simultaneously.
(TIF)

Table S1 The lineage, temporal and spatial distribution of 1178 H9 subtype AIVs reported to GenBank.
(DOCX)

Table S2 Genetic distances between different levels of H9 lineages.
(DOCX)

Table S3 Hemagglutination titers of 76 H9 viruses against goose RBCs untreated or treated with α2,3-specific sialidase.
(DOCX)
