# Supplementary figures and images for: Chinese and Global Distribution of H9 Subtype Avian Influenza Viruses
Source: PLoS One. 2012 Dec 21;7(12):e52671. doi: 10.1371/journal.pone.0052671 (PMC3528714; doi:10.1371/journal.pone.0052671)

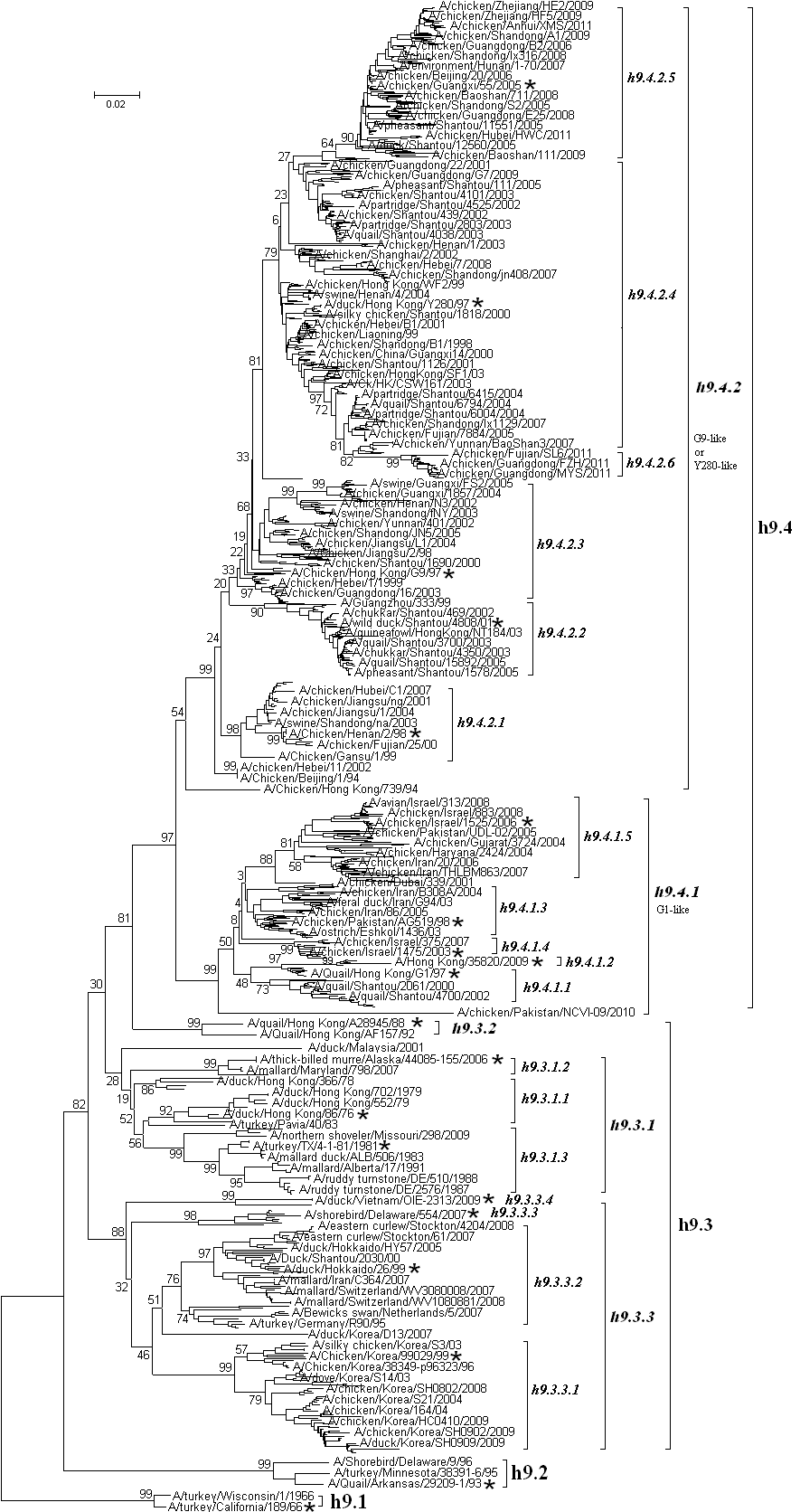

Supplement: Figure S1 — Small version of the phylogenetic tree of 1178 H9 subtype AIVs based on HA1 subunit of the viral hemagglutinin gene sequences. Lineage designations are to the right of relevant branches, and bootstrap values are at relevant nodes. The representative virus of each lineage is marked with an asterisk. This figure is too large, and it actual size can be viewed using Windows-Picture-&-Fax Viewer via pressing the keys “Ctrl” and “A” simultaneously. (TIF) [file pone.0052671.s001.tif]

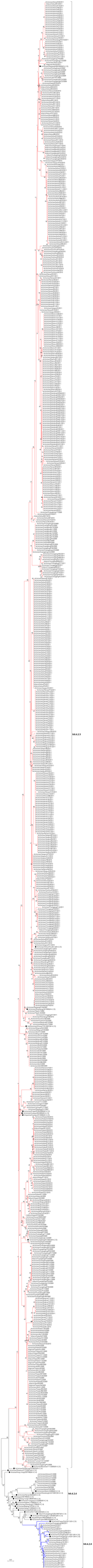

Supplement: Figure S3 — Phylogenetic distribution of 800 H9 subtype AIVs based the sequences of the HA1 subunit of the viral hemagglutinin gene. The representative virus of each lineage is marked with an asterisk. Lineage designations are to the right of relevant branches, and bootstrap values are at relevant nodes. This figure is too large, and it actual size can be viewed using Windows-Picture-&-Fax Viewer via pressing the keys “Ctrl” and “A” simultaneously. (TIF) [file pone.0052671.s003.tif]
