# Supplementary material for: Chinese and Global Distribution of H9 Subtype Avian Influenza Viruses
Source: PLoS One. 2012 Dec 21;7(12):e52671. doi: 10.1371/journal.pone.0052671 (PMC3528714; doi:10.1371/journal.pone.0052671)
Supplement: Table S1 — The lineage, temporal and spatial distribution of 1178 H9 subtype AIVs reported to GenBank. (DOCX) [file pone.0052671.s004.docx]

**Table S1.** The lineage, temporal and spatial distribution of 1,178 H9 subtype AIVs reported to GenBank ^a^

| Lineage | Before 1990 | In the 1990s | In 2000-2006 | After 2006 |
| --- | --- | --- | --- | --- |
| h9.1 | **USA(2)** |  |  |  |
| h9.2 |  | **USA(3)** |  |  |
| h9.3.1.1 | *HK(11)* **NZL(1)** |  |  |  |
| h9.3.1.2 |  |  | **USA(3)** | **USA(2)** |
| h9.3.1.3 | **USA(10) CAN(4)** | **CAN(2)** | **KOR(1)** | **USA(2)** |
| h9.3.3.1 |  | **KOR(6)** | **KOR(23)** | **KOR(33)** |
| h9.3.3.2 |  | **GER(5) IRL(2) JPN(4) RSA(1)** *HK(1)* | **KOR(1) NED(4) JPN(4) RUS(2) VIE(2) MGL(1)** *GD(4)* | **AUS(8) NED(1) JPN(1) SUI(8) AUT(1) IRI(4) POR(1) RSA(1) ZAM(1)** |
| h9.3.3.3 |  |  | **USA(9)** | **USA(1)** |
| h9.3.3.4 |  |  |  | **VIE(3)** |
| h9.3.2 | *HK(1)* | *HK(1)* |  |  |
| h9.4.1.1 |  | *HK(10)* | *GD(5)* | *HK(2)* |
| h9.4.1.2 |  |  | *GD(31)* |  |
| h9.4.1.3 |  | **GER(1) JPN(2) IRI(8) KSA(2) PAK(4) UAE(1)** | **ISR(11) IRI(21) KSA(1) UAE(8)** | **IRI(1)** |
| h9.4.1.4 |  |  | **ISR(19) IRI(1)** | **ISR(2)** |
| h9.4.1.5 |  |  | **ISR(4) IRI(11) KSA(3) PAK(6) IND(12)** | **ISR(25) LIB(2) IRI(20) PAK(6) IND(1)** |
| h9.4.2.1 |  | *GS(1) NX(1) AH(1) JS(2) HE(2) SD(1) HA(2) BJ(1) SH(2)* **JPN(1)** | *FJ(2) YN(1) JS(9) HE(2) SD(3) HA(1) ZJ(1) SH(1) GX(1) NM(1)* | *HB(1)* |
| h9.4.2.2 |  | *GD(1)* | *GD(74) HK(4) HN(1)* |  |
| h9.4.2.3 |  | *GD(9) YN(2) HK(7) JS(5) HE(2) SD(3) BJ(2) GX(4) SC(1) HJ(1) TJ(2)* | *GD(9) YN(8) AH(2) JS(10) HE(1) SD(14) HA(5) BJ(2) SH(11) GX(20) HN(1) JX(1) HJ(2)* **JPN(4)** | *JS(1) SD(3) HA(2) GX(3)* |
| h9.4.2.4 |  | *GD(4) HK(9) GS(1) NX(1) HE(3) SD(4) HA(4) BJ(4) SH(1) GX(1) LN(1)* | *GD(140) FJ(9) YN(5) HK(24) JS(3) HE(7) SD(5) HA(13) HB(1) ZJ(1) BJ(2) SH(3) GX(6) JX(6) SC(1) HJ(1) TJ(1) LN(2) JL(1) CN(1)* **JPN(6)** | *GD(3) FJ(3) YN(9) HE(6) SD(22) HA(4) GX(4) SC(3) LN(1) JL(1)* |
| h9.4.2.5 |  | *SD(1)* | *GD(13) FJ(9) SD(3) HA(1) BJ(4) GX(9) HN(7)* | *GD(13) FJ(3) YN(17) XZ(3) HK(1) AH(9) JS(11) HE(4) SD(15) HA(1) HB(6) ZJ(19) GX(3) HN(10) SC(4) HI(1)* |
| h9.4.2.6 |  |  |  | *GD(24) FJ(1) GX(1)* |

^a^ The numbers of viruses are given in the parentheses after the relevant names of countries or provinces in China; country names are given in bold and abbreviated as follows: AUS=Australia, AUT=Austria, CAN=Canada, GER=Germany,IND=India, IRI=Iran, IRL=Ireland, ISR=Israel, JPN=Japan, KOR=Korea, KSA=Saudi Arabia, LIB=Lebanon, MGL=Mongolia, NED=Netherlands, NZL=New Zealand, PAK=Pakistan, POR=Portugal, RSA=South Africa, RUS=Russia, SUI=Switzerland, UAE=United Arab Emirates, USA=United States of America, VIE=Vietnam, ZAM=Zambia; province names of China are given in italics and abbreviated as follows: AH=Anhui, BJ=Beijing, FJ=Fujian, GD=Guangdong, GS=Gansu, GX=Guangxi, HA=Henan, HB=Hubei, HE=Hebei, HI=Hainan, HJ=Heilongjiang, HK=Hong Kong, HN=Hunan, JL=Jilin, JS=Jiangsu, JX=Jiangxi, LN=Liaoning, NM=Inner Mongolia, NX=Ningxia, SC=Sichuan, SD=Shandong, SH=Shanghai, TJ=Tianjin, XZ=Tibet, YN=Yunnan, ZJ=Zhejiang, CN=unknown provinces in China.
