# Supplementary material for: Chinese and Global Distribution of H9 Subtype Avian Influenza Viruses
Source: PLoS One. 2012 Dec 21;7(12):e52671. doi: 10.1371/journal.pone.0052671 (PMC3528714; doi:10.1371/journal.pone.0052671)
Supplement: Table S2 — Genetic distances between different levels of H9 lineages. (DOCX) [file pone.0052671.s005.docx]

**Table S2. Genetic distances between different levels of H9 lineages.**

**1. Genetic distances between the primary lineages (20.00% - 24.39%, Mean= 22.02%)**

| **Lineages** | **Distances** |
| --- | --- |
| h9.1 vs h9.2 | 22.29% |
| h9.1 vs h9.3 | 21.23% |
| h9.1 vs h9.4 | 22.64% |
| h9.2 vs h9.3 | 21.56% |
| h9.2 vs h9.4 | 24.39% |
| h9.3 vs h9.4 | 20.00% |

**2. Genetic distances between the secondary lineages (11.93%-17.27%, mean=14.69%)**

| **Lineages** | **Distances** |
| --- | --- |
| h9.3.1 vs h9.3.2 | 11.93% |
| h9.3.1 vs h9.3.3 | 14.88% |
| h9.3.2 vs h9.3.3 | 17.27% |
| h9.4.1 vs h9.4.2 | 14.67% |

**3. Genetic distances between the tertiary lineages (5.81%-18.28%, mean=10.08%)**

| **Lineages** | **Distances** |
| --- | --- |
| h9.3.1.1 vs h9.3.1.2 | 10.08% |
| h9.3.1.1 vs h9.3.1.3 | 9.94% |
| h9.3.1.2 vs h9.3.1.3 | 12.38% |
| h9.3.3.1 vs h9.3.3.2 | 14.17% |
| h9.3.3.1 vs h9.3.3.3 | 15.30% |
| h9.3.3.1 vs h9.3.3.4 | 18.28% |
| h9.3.3.2 vs h9.3.3.3 | 11.27% |
| h9.3.3.2 vs h9.3.3.4 | 13.06% |
| h9.3.3.3 vs h9.3.3.4 | 14.64% |
| h9.4.1.1 vs h9.4.1.2 | 6.15% |
| h9.4.1.1 vs h9.4.1.3 | 6.58% |
| h9.4.1.1 vs h9.4.1.4 | 8.08% |
| h9.4.1.1 vs h9.4.1.5 | 10.24% |
| h9.4.1.2 vs h9.4.1.3 | 6.44% |
| h9.4.1.2 vs h9.4.1.4 | 7.64% |
| h9.4.1.2 vs h9.4.1.5 | 10.15% |
| h9.4.1.3 vs h9.4.1.4 | 5.81% |
| h9.4.1.3 vs h9.4.1.5 | 9.15% |
| h9.4.1.4 vs h9.4.1.5 | 10.35% |
| h9.4.2.1 vs h9.4.2.2 | 8.00% |
| h9.4.2.1 vs h9.4.2.3 | 6.23% |
| h9.4.2.1 vs h9.4.2.4 | 8.10% |
| h9.4.2.1 vs h9.4.2.5 | 10.49% |
| h9.4.2.1 vs h9.4.2.6 | 11.50% |
| h9.4.2.2 vs h9.4.2.3 | 8.44% |
| h9.4.2.2 vs h9.4.2.4 | 9.92% |
| h9.4.2.2 vs h9.4.2.5 | 12.92% |
| h9.4.2.2 vs h9.4.2.6 | 12.95% |
| h9.4.2.3 vs h9.4.2.4 | 6.70% |
| h9.4.2.3 vs h9.4.2.5 | 9.65% |
| h9.4.2.3 vs h9.4.2.6 | 10.29% |
| h9.4.2.4 vs h9.4.2.5 | 7.75% |
| h9.4.2.4 vs h9.4.2.6 | 7.64% |
| h9.4.2.5 vs h9.4.2.6 | 12.35% |
