# Supplementary material for: Chinese and Global Distribution of H9 Subtype Avian Influenza Viruses
Source: PLoS One. 2012 Dec 21;7(12):e52671. doi: 10.1371/journal.pone.0052671 (PMC3528714; doi:10.1371/journal.pone.0052671)
Supplement: Table S3 — Hemagglutination titers of 76 H9 viruses against goose RBCs untreated or treated with α2,3-specific sialidase. (DOCX) [file pone.0052671.s006.docx]

Table S3. Hemagglutination titers of 76 H9 viruses against goose RBCs untreated or treated with α2,3-speciﬁc sialidase ^a^.

| Virus | Lineage | Amino acid residue at position 234 | Titers (untreated) | Titers (treated) |
| --- | --- | --- | --- | --- |
| A/chicken/Chongqing/K3/2011 | h9.4.2.5 | Leu | 1024 | 1024 |
| A/chicken/Hainan/J6/2011 | h9.4.2.5 | Leu | 1024 | 1024 |
| A/chicken/Hainan/J7/2011 | h9.4.2.5 | Leu | 512 | 512 |
| A/chicken/Jiangsu/B13/2011 | h9.4.2.5 | Leu | 256 | 256 |
| A/chicken/Shandong/WE41/2011 | h9.4.2.5 | Leu | 256 | 256 |
| A/chicken/Shandong/J9/2011 | h9.4.2.5 | Leu | 128 | 128 |
| A/chicken/Anhui/A29/2011 | h9.4.2.5 | Leu | 128 | 128 |
| A/chicken/Shandong/WG18/2011 | h9.4.2.5 | Leu | 2048 | 1024 |
| A/chicken/Jiangsu/B30/2011 | h9.4.2.5 | Leu | 1024 | 512 |
| A/chicken/Shandong/WE37/2011 | h9.4.2.5 | Leu | 512 | 256 |
| A/chicken/Hainan/J19/2011 | h9.4.2.5 | Leu | 256 | 128 |
| A/chicken/Hainan/J4/2011 | h9.4.2.5 | Leu | 64 | 32 |
| A/chicken/Chongqing/K9/2011 | h9.4.2.5 | Leu | 2048 | 512 |
| A/chicken/Ningxia/184/2011 | h9.4.2.6 | Leu | 1024 | 256 |
| A/chicken/Shanxi/B11/2011 | h9.4.2.5 | Leu | 1024 | 256 |
| A/chicken/Henan/H15/2011 | h9.4.2.5 | Leu | 1024 | 256 |
| A/chicken/Shanxi/F4/2011 | h9.4.2.5 | Leu | 1024 | 256 |
| A/chicken/Chongqing/K4/2011 | h9.4.2.5 | Leu | 1024 | 256 |
| A/chicken/Hainan/I22/2011 | h9.4.2.6 | Leu | 512 | 128 |
| A/chicken/Chongqing/J24/2011 | h9.4.2.5 | Leu | 256 | 64 |
| A/chicken/Shandong/H23/2011 | h9.4.2.5 | Leu | 256 | 64 |
| A/chicken/Ningxia/182/2011 | h9.4.2.6 | Leu | 256 | 64 |
| A/chicken/Chongqing/J29/2011 | h9.4.2.5 | Leu | 256 | 64 |
| A/chicken/Shandong/WE27/2011 | h9.4.2.5 | Leu | 256 | 64 |
| A/chicken/Shanxi/I13/2011 | h9.4.2.5 | Leu | 256 | 64 |
| A/chicken/Anhui/K12/2011 | h9.4.2.5 | Leu | 1024 | 128 |
| A/chicken/Hainan/J3/2011 | h9.4.2.5 | Leu | 512 | 64 |
| A/chicken/Anhui/E15/2011 | h9.4.2.5 | Leu | 512 | 64 |
| A/chicken/Hainan/J18/2011 | h9.4.2.5 | Leu | 512 | 64 |
| A/chicken/Shanxi/J7/2011 | h9.4.2.5 | Leu | 512 | 64 |
| A/chicken/Anhui/K3/2011 | h9.4.2.5 | Leu | 256 | 32 |
| A/chicken/Jiangsu/D10/2011 | h9.4.2.5 | Leu | 256 | 32 |
| A/chicken/Anhui/A21/2011 | h9.4.2.5 | Leu | 256 | 32 |
| A/chicken/Shandong/YB06/2006 | h9.4.2.5 | Gln | 256 | 32 |
| A/chicken/Anhui/K30/2011 | h9.4.2.5 | Leu | 128 | 16 |
| A/chicken/Henan/J17/2011 | h9.4.2.5 | Leu | 128 | 16 |
| A/chicken/Chongqing/A56/2011 | h9.4.2.5 | Leu | 2048 | 128 |
| A/chicken/Ningxia/31/2011 | h9.4.2.5 | Leu | 512 | 32 |
| A/chicken/Anhui/K9/2011 | h9.4.2.5 | Leu | 512 | 32 |
| A/chicken/Henan/J14/2011 | h9.4.2.5 | Leu | 512 | 32 |
| A/chicken/Henan/G10/2011 | h9.4.2.5 | Leu | 256 | 16 |
| A/chicken/Henan/G5/2011 | h9.4.2.5 | Leu | 256 | 16 |
| A/chicken/Chongqing/A52/2011 | h9.4.2.5 | Leu | 2048 | 64 |
| A/chicken/Shanxi/F28/2011 | h9.4.2.5 | Leu | 1024 | 32 |
| A/chicken/Jiangsu/B18/2011 | h9.4.2.5 | Leu | 1024 | 32 |
| A/chicken/Henan/F22/2011 | h9.4.2.5 | Leu | 1024 | 32 |
| A/chicken/Shanxi/I21/2011 | h9.4.2.5 | Leu | 1024 | 32 |
| A/chicken/Anhui/I28/2011 | h9.4.2.5 | Leu | 1024 | 32 |
| A/chicken/Ningxia/99/2011 | h9.4.2.5 | Leu | 512 | 16 |
| A/chicken/Henan/F28/2011 | h9.4.2.5 | Leu | 4096 | 64 |
| A/chicken/Shandong/WG10/2011 | h9.4.2.5 | Leu | 1024 | 16 |
| A/duck/Chongqing/B36/2011 | h9.4.2.5 | Leu | 512 | 8 |
| A/chicken/Chongqing/K7/2011 | h9.4.2.5 | Leu | 1024 | 8 |
| A/chicken/Anhui/D21/2011 | h9.4.2.5 | Leu | 512 | 4 |
| A/chicken/Ningxia/189/2011 | h9.4.2.5 | Leu | 256 | 2 |
| A/chicken/Anhui/K4/2011 | h9.4.2.5 | Leu | 2048 | 1 |
| A/chicken/Shandong/BZ02/2002 | h9.4.2.3 | Gln | 512 | 1 |
| A/chicken/Anhui/E24/2011 | h9.4.2.5 | Leu | 512 | 1 |
| A/chicken/Henan/F15/2011 | h9.4.2.5 | Leu | 512 | 1 |
| A/chicken/Shanxi/J21/2011 | h9.4.2.5 | Leu | 512 | 1 |
| A/chicken/Chongqing/K10/2011 | h9.4.2.5 | Leu | 256 | 1 |
| A/chicken/Chongqing/G11/2011 | h9.4.2.5 | Leu | 128 | 1 |
| A/chicken/Henan/I25/2011 | h9.4.2.5 | Leu | 128 | 1 |
| A/chicken/Ningxia/A2/2011 | h9.4.2.5 | Leu | 128 | 1 |
| A/chicken/Henan/F9/2011 | h9.4.2.5 | Leu | 128 | 1 |
| A/chicken/Henan/F13/2011 | h9.4.2.5 | Leu | 128 | 1 |
| A/chicken/Ningxia/47/2011 | h9.4.2.5 | Leu | 64 | 1 |
| A/chicken/Anhui/D19/2011 | h9.4.2.5 | Leu | 64 | 1 |
| A/chicken/HenanF1/2011 | h9.4.2.5 | Leu | 64 | 1 |
| A/chicken/Henan/G22/2011 | h9.4.2.5 | Leu | 64 | 1 |
| A/chicken/Henan/F3/2011 | h9.4.2.5 | Leu | 64 | 1 |
| A/chicken/Anhui/J9/2011 | h9.4.2.5 | Leu | 64 | 1 |
| A/chicken/Anhui/F29/2011 | h9.4.2.5 | Leu | 64 | 1 |
| A/chicken/Henan/F5/2011 | h9.4.2.5 | Leu | 64 | 1 |
| A/chicken/Henan/F18/2011 | h9.4.2.5 | Leu | 64 | 1 |
| A/chicken/Henan/E21/2011 | h9.4.2.5 | Leu | 64 | 1 |

^a^ The lineages other than h9.4.2.5 and the amino acid residue of Gln at position 234 are in shadow.
